# Assessment of parameters reflecting the reactivity of the autonomic nervous system of Polish firefighters on the basis of a test in a smoke chamber

**DOI:** 10.3389/fpubh.2024.1426174

**Published:** 2024-07-19

**Authors:** Łukasz Dudziński, Łukasz Czyżewski, Mariusz Panczyk

**Affiliations:** ^1^Department of Medical Rescue, John Paul II University in Biała Podlaska, Biała Podlaska, Poland; ^2^Geriatric Nursing Facility, Medical University of Warsaw, Warsaw, Poland; ^3^Faculty of Health Sciences, Medical University of Warsaw, Warsaw, Poland

**Keywords:** psychophysical burden on firefighters, smoke chamber test, rescue and firefighting operations, sinus rhythm variability, health risk

## Abstract

**Objective:**

Measurement and analysis of heart rate variability in a population of professional firefighters based on heart rate (RR) recording. Assessment based on a smoke chamber test in correlation with age, length of service, body mass index.

**Materials and methods:**

The smoke chamber test for the officers of the State Fire Service (SFS) is aimed at improving the skills and techniques of working in special clothing and in a respiratory protection set (RPS) under high psychophysical burden. The study was divided into 3 stages: 1. measurement of parameters at rest – sitting position for 5 min, 2. measurement of parameters during the firefighter’s activity, effort related to the training path and the test in the smoke chamber, indefinite time (different for each firefighter), 3. measurement of parameters at rest after exercise – sitting position for 5 min. Each firefighter included in the study had fitted onto his chest a Polar H10 band with a sensor (size XXL) that measures parameters HR, HRV (sensor connected via Bluetooth to an application on the phone of a person controlling the test).

**Results:**

The study involved 96 firefighters aged 19–45 (Mean 27.9; SD 7.4), with 1–19 years of service (Mean 5.2; SD 4.6). The study included 75 firefighters who completed the entire activity and their results were recorded completely in a way that allowed for analysis and interpretation. Results of 17 firefighters were selected (parameters describing HRV changes was carried out, which are important from the authors’ experience: RMSSD, HF ms^2^, DFA α1).

**Conclusion:**

The presence of excessive body weight did not affect HR parameters, which may be related to the limited possibilities of using the BMI index among people with high muscle mass. Longer work experience has a health-promoting effect on heart rate values through increased adaptation of the circulatory system to increased effort and stress. HRV parameter and ANS activity have a wide range of clinical applications, in addition to monitoring health status in the course of diseases, ANS activity can be analyzed in correlation with occupational risk factors.

## 1 Introduction

Firefighters are a uniformed formation, which in its statutory activities is tasked with saving human life, health, rescuing animals, rescuing property, counteracting natural disasters, industrial accidents, and catastrophes. Firefighters often operate in the danger zone, where other services (National Emergency Medical Services, Police) cannot stay, and evacuation of injured people outside the danger zone requires the firefighter to be in good health and in good physical condition (1, 2).

Firefighters undergo health examinations every year. In addition, they undergo a fitness and performance assessment during a smoke chamber test. Each firefighter is required to go through the smoke chamber path with the frequency of the test at least once every three years in accordance with the guidelines of the National Headquarters of the State Fire Service (NHSFS). In the smoke chamber, firefighters’ resistance to psychophysical burden is tested in conditions similar to real operations. During tests and improvement exercises, orientation and the ability to move in smoky conditions and confined spaces are checked. A rescuer wearing a breathing apparatus overcomes various obstacles in conditions of increased temperature, noise and light effects. The test in the chamber is physically burdensome for firefighters, additional burden is the increased temperature in the chamber and full uniform, as well as the weight of the air cylinder carried on the back during the test (3, 4).

Heart rate variability (HRV) is a non-invasive, objective and validated method for assessing the autonomic nervous system. The analysis of differences in the time interval between successive peaks of the R waves of the QRS complex (in electrocardiogram) has a wide range of clinical applications. The measurement of this RR interval is used to calculate heart rate variability. Heart rate variability is believed to reflect modulation of sinus node automaticity by the sympathetic and parasympathetic parts of the autonomic nervous system. Numerous studies indicate clinical use of cardiac rhythm variability in determining the prognosis after myocardial infarction and the risk of sudden cardiac death, disease prevention, and even autonomic function in the post-COVID-19 period (5–7).

The parameter can be used as a predictor of human health, as heart rhythm is modulated by a wide range of physiological processes. The availability of modern, high-quality sensors and the low data rate of heart rate signals make HRV easy to measure, transmit, store and process. However, there are also significant obstacles preventing the wider use of this technology. HRV signals are non-stationary, non-linear, and resemble noise to the human eye (8).

One of the methods of measuring HRV is to use a band with a sensor placed on the chest. The sensor is provided with a transmitter connected via Bluetooth to an application on a phone or other mobile device that allows access to the raw heart rate data (RR intervals), from which the HRV parameter can be calculated. So far, HRV analysis has been used mainly in cardiology, but now it is used in sports and in monitoring the workload of uniformed services (9).

The assessment of the autonomic nervous system (ANS) in firefighters using HRV analysis in firefighters has been carried out in recent years in several research projects outside Poland. The Gendron study concerned the dependence of HRV in the period preceding firefighting activities. Fire extinguishing action is modulated by different work cycles (simulated extinguishing actions in correlation with the passive rest period) (10).

Buecher undertook work on classifying the cognitive and physical stress of firefighters based on heart rate variability parameters. The study was carried out on a group of 85 firefighters in a cage maze, which is methodologically similar to the test in a smoke chamber in our own study (11).

Measurement and analysis of heart rate variability in a population of professional firefighters based on heart rate (RR) recording. Assessment based on a smoke chamber test in correlation with age, length of service, body mass index.

## 2 Materials and methods

### 2.1 Design

The smoke chamber test for the officers of the State Fire Service (SFS) is aimed at improving the skills and techniques of working in special clothing and in a respiratory protection set (RPS) under high psychophysical burden. Elements that burden and make it difficult to complete the test include limited visibility (darkening and smoke), limited space, increased temperature and noise.

The physical exertion caused by the exercises provided for the path (squeezing through narrow spaces, moving on your knees, crawling) reflects the realism of real rescue and firefighting operations. The smoke chamber test was carried out at the Training Centers of the Provincial Headquarters Lublin of the State Fire Service in Poland.

**Figure 1:**
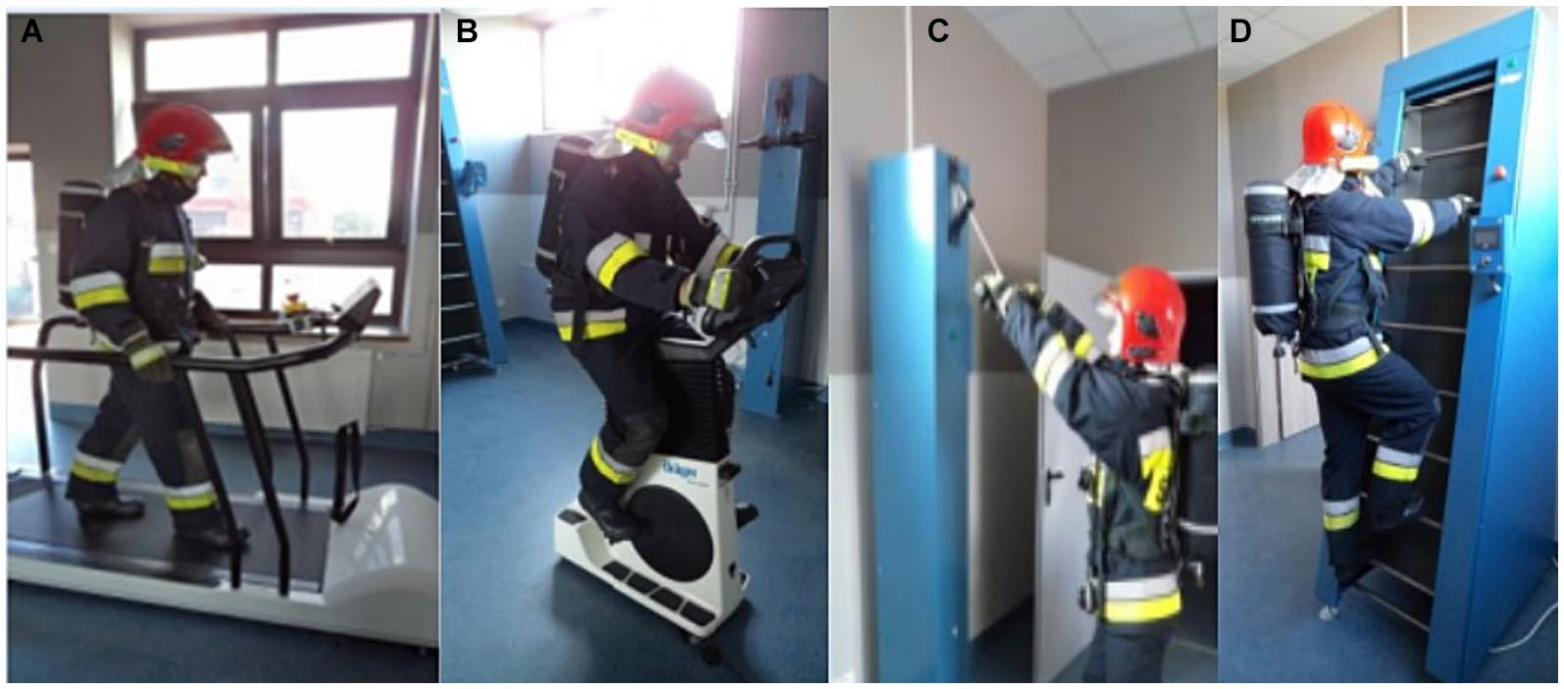
Training path – subsequent stages of fatigue before the smoke chamber. Source: State Fire Service: Smoke Chamber Test Regulations. **(A)** Belt ergometer (walking on a treadmill)- 6 min activity- speed depending on the firefighter’s age, **(B)** bicycle ergometer (stationary bike with a load)- 1 min with a load depending on the firefighter’s age category, **(C)** pull-up hammer (raising and lowering a specific weight)- the number of pull-ups from 10 to 20 – depends on the age category of the firefighter, **(D)** endless ladder (moving ladder)- 2 min – speed depending on the firefighter’s age (12).

**Figure 2:**
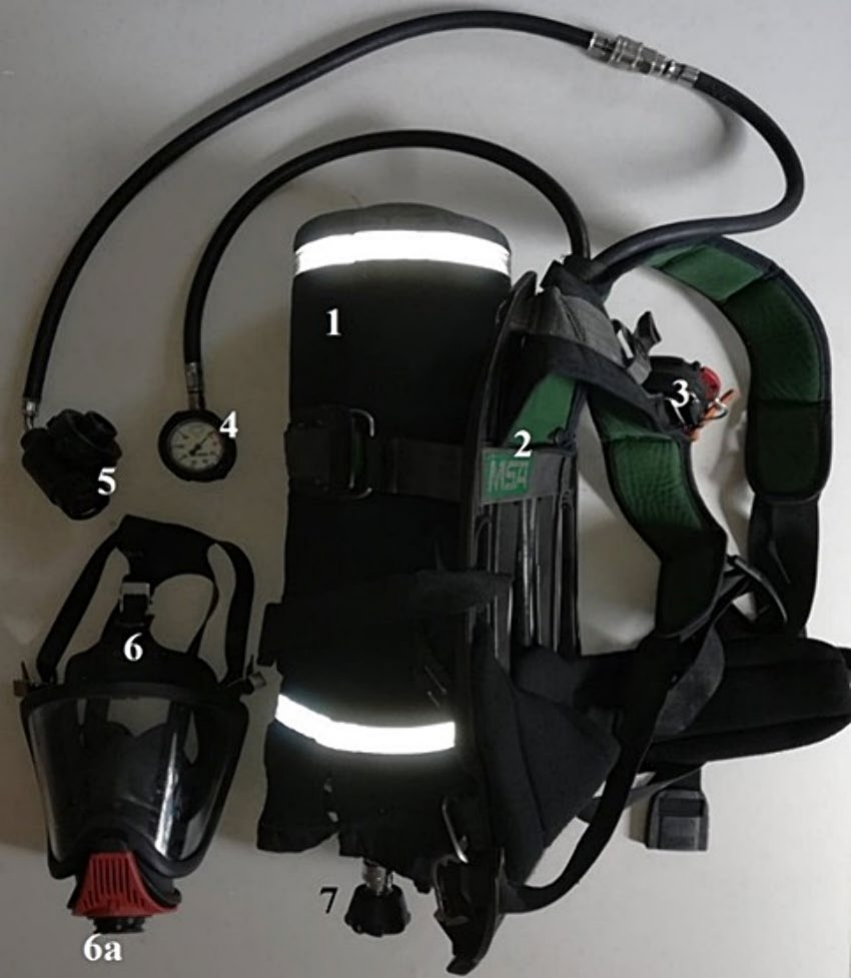
Respiratory protection apparatus. Source: Authors’ private archive.

Before proceeding to the actual part of the test, firefighters undergo a stress test (Figure 1). The test was carried out in special clothing, gloves, helmet, firefighter footwear with the RPS on, but not connected (the breathing apparatus is on the back without a mask attached). The effect of fatigue in the smoke chamber is increased by the burden on the respiratory tract of the connected RPS set and the burden associated with carrying an air cylinder on the back (Figure 2). Breathing through the RPS, even without engaging in any activities, i.e., while standing still, burdens organism through increased respiratory effort. During the test, firefighters are dressed in full special (combat) clothing (12).

The kit includes: 1. compressed air cylinder, 2. frame with shoulder straps and hip belt, 3. immobility indicator, 4. manometer indicating the air filling level of the cylinder, 5. breathing apparatus, 6. mask with panoramic glass, 6a. inhalation valve – connection point for the mask with the breathing apparatus, 7. cylinder valve.

### 2.2 Study procedure

The study was divided into 3 stages:

- Stage 1 – measurement of parameters at rest – sitting position for 5 min.
- Stage 2 – measurement of parameters during the firefighter’s activity, effort related to the training path and the test in the smoke chamber, indefinite time (different for each firefighter)- this part included the time of the training track (Figure 1), the time of moving between stations, and the time of the actual chamber. According to the regulations, the passage time of the smoke chamber is limited to the capacity of 1 compressed air cylinder. The condition for passing the training path is that the test participant completes it using one breathing apparatus.
- Stage 3 – measurement of parameters at rest after exercise – sitting position for 5 min.

In the statistical analysis of HR and HRV, the above 3 stages are called: PRE, DURING, POST. Each firefighter included in the study had fitted onto his chest a Polar H10 band with a sensor (size XXL is intended for chest circumference in the range of 70–115 cm) that measures parameters (HR, HRV). At each stage, the band was connected via Bluetooth to an application on the phone of a person controlling the test, which allowed access to the raw heart rate data (RR intervals), from which the HRV parameter was calculated.

Polar H10 sensors are new and have been used by firefighters since new, they did not require calibration, approved for sale in Poland with a wide range of applications in many fields of sports, calibrated with a mobile device through a dedicated application from the manufacturer’s online website. The smoke chamber is permitted by the regulation of the Minister of Internal Affairs and Administration of September 16, 2008 on detailed health and safety conditions for firefighters of the State Fire Service (Journal of Laws No. 180, item 1,115). based on the opinion of the Scientific and Research Center of the State Fire Service. This is a standardized test for the whole of Poland according to the PN EN 60439–1 and PN EN 60335–1 standards. The regulations assume that before starting the test the chamber staff conducts an initial instruction, which includes a discussion of: the purpose of the exercises, the structure of the chamber, the organization and sequence of the exercises, the devices and tasks to be performed on the path, the health and safety rules during the exercises.

The firefighter performing the test signs a declaration that he has not consumed alcohol or other psychoactive substances in the last 48 h, and has not performed any activities involving long-term physical exertion in the last 24 h; in addition, the firefighter must have a current medical certificate and occupational health and safety training.

### 2.3 Smoke chamber test protocol

The training path is covered in special clothing, a helmet, special footwear, special gloves, a balaclava and respiratory protection equipment. The mask is connected to the breathing apparatus and placed on the face. The minimum length of the training path, made on two levels (crawling level, walking level) – 24 m. The path is made of modular elements, forming a square in the base with a side length of 750÷800 mm, connected to each other and forming one whole. The height of the crawling level – 0.87÷0.9 m.

The lighting in the chamber is identical for all participants: the number of lamps installed in the chamber compartments must allow for obtaining the light intensity level in accordance with the Polish Standard PN-EN 12464-1.

The authors do not know what the optical density of the smoke is inside the chamber. The temperature in the upper part of the chamber increased to 70°C. For the authors, it is important that all participants have the same conditions. During the test/improvement exercises, the participant is under constant supervision of the chamber staff and devices monitoring the course of the test/improvement exercises. The conditions in the chamber are difficult, the firefighter with the sensor is moving dynamically.

There is a large amount of metal obstacles–manhole – installed in place of the floor element with a hole with a diameter of 600 mm, closed with a hinged cover, enabling passage between levels – number adapted to the route of the path, short pipe – made of plastic, length 200 mm, diameter 600 mm, with a frame to be placed on the path, long pipe – made of plastic, one module long, diameter 600 mm, with two frames to be placed on the path, grate (door) large sliding – an obstacle made of grate, installed inside the walking level, After moving which there is a passage of approx. 600 mm to the next segment – 1 piece, small sliding grate (door) – an obstacle made of grate, installed inside the crawling level, after which after which it remains to move approx. 600 mm to the next segment, large horizontally narrowed grate – an obstacle made of a grate with horizontal division, half filled with grate, mounted inside the walking level.

There are also electronic devices: camera with a built-in infrared illuminator with a resolution of min. 480 lines and visibility min. 20 m, for observation in the light and in the dark in the presence of smoke, with a mounting base and a protective barrier. The camera is placed in a place and in a way that ensures observation of the entire chamber space, controlled from the control panel. The number of cameras adapted to the layout of the training path, thermal imaging camera (does not require cooling), with a set of accessories for observing exercises in the presence of smoke, placed in a place and in a way that ensures observation of the entire space of the chamber, controlled from the control panel, also fogging and heating devices.

All chambers in Poland have the same procedure, dimensions, structure, and training path - which ensures repeatability and standard of the test from the technical side (2, 12).

### 2.4 Data collection procedure details

- the Polar H10 sensor sent the test record (date, time, duration of the test, obtained parameters) to a virtual individual profile (set up by the authors of the study) on the Polar manufacturer’s website: www.flow.polar.com.
- the results were exported to an MSExcel file with the data provided by the software.
- raw results from the Ms. Excel file were processed with Kubios software.

Stress factors in the chamber environment: temperature, smoke density and noise level in the smoke chamber - are not known to the authors. These are parameters standardized for the whole of Poland and generated automatically by the smoke chamber software.

For the purposes of statistical calculations, the following data were obtained from each firefighter:

- age.
- seniority.
- another test in the smoke chamber.
- height and weight (BMI).

**Figure 3:**
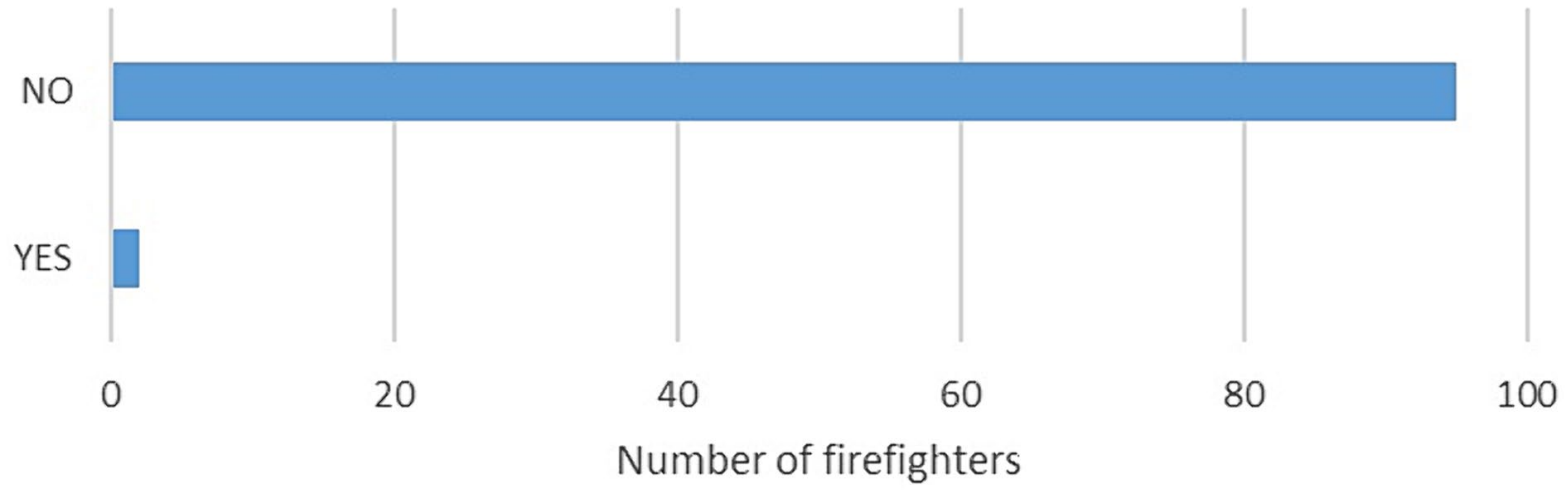
Assessment of work (effort with the band). Closed question: Was the smoke chamber test with the Polar H10 band on the chest difficult?

After leaving the chamber, each firefighter was asked whether the PolarH10 band hindered his activity – a closed question: yes/no (Figure 3).

#### 2.4.1 Ethical considerations

The described cases are fully anonymous, the analysis is in accordance with the principles of the Declaration of Helsinki and the GDPR regulations. All firefighters included in the study gave informed consent to participate. The consent of the bioethics committee of the XXX XXX University No. 19/2022 of 19.12.2022 was obtained for the implementation of the above project. In addition, the consent of the Provincial Commander of the State Fire Service was obtained for the implementation of the practical part of the study.

#### 2.4.2 Characteristics of the study group

Firefighters from the district and municipal headquarters of the State Fire Service in the Lublin Voivodeship took part in the study. The study group consists of firefighters holding various official positions in the combat division (commander, rescuer, driver). The study was anonymous and voluntary, of which the participants were informed. At the same time, the subjects were informed that they could stop participating in the study at any stage.

### 2.5 Statistical analysis

Results concerning quantitative variables (HR min, HR max, HR mean) were presented as average values ± standard deviation. Results (RMSSD, HF ms^2^, DFA α1) were presented as median and interquartile range (IQR). The Kolmogorov–Smirnov test was used to assess the normality of the distributions.

In the comparative analysis of the stages of study (Pre, During and Post) depending on: heart rate (HR) min, HR max, HR mean, RR interval differences (RMSSD), high frequent (HF) ms^2^ and detrended fluctuation analysis (DFA) α1, Friedman ANOVA was used.

For results presented as average values ± standard deviation, groups were discretized according to Mean-based discretization. For results presented as median and interquartile range (IQR), groups were discretized according to Standard median splits. Proportions in groups divided by age, BMI, employment experience and activity were assessed with a Chi-squared test. Statistica 13 software (StatSoft Inc., Tulsa, OK) was used for the statistical analysis.

A significance level of *p* < 0.05 was adopted.

## 3 Results

The study involved 96 firefighters aged 19–45 (Mean 27.9; SD 7.4), with 1–19 years of service (Mean 5.2; SD 4.6). The study included 75 firefighters who completed the entire activity and their results were recorded completely in a way that allowed for analysis and interpretation (Table 1).

**Table 1:** General characteristics of the study group.

	N	M	SD	Median	IQR/2	Q1	Q3	Min	Max
Age [yr.]	75	27.95	7.49	26.00	6.50	22.00	35.00	18.00	45.00
Seniority [yr.]	75	5.25	4.69	4.00	3.50	1.00	8.00	1.00	19.00
Activity [min.]	75	39.69	14.89	38.00	9.00	31.00	49.00	8.00	91.00
BMI [kg/m^2^]	75	25.37	2.23	25.59	1.40	23.92	26.73	20.06	30.96

M, mean; SD, standard deviation; IQR/2, semi-interquartile range; Q1, lower quartile; Q3, upper quartile.

**Figure 4:**
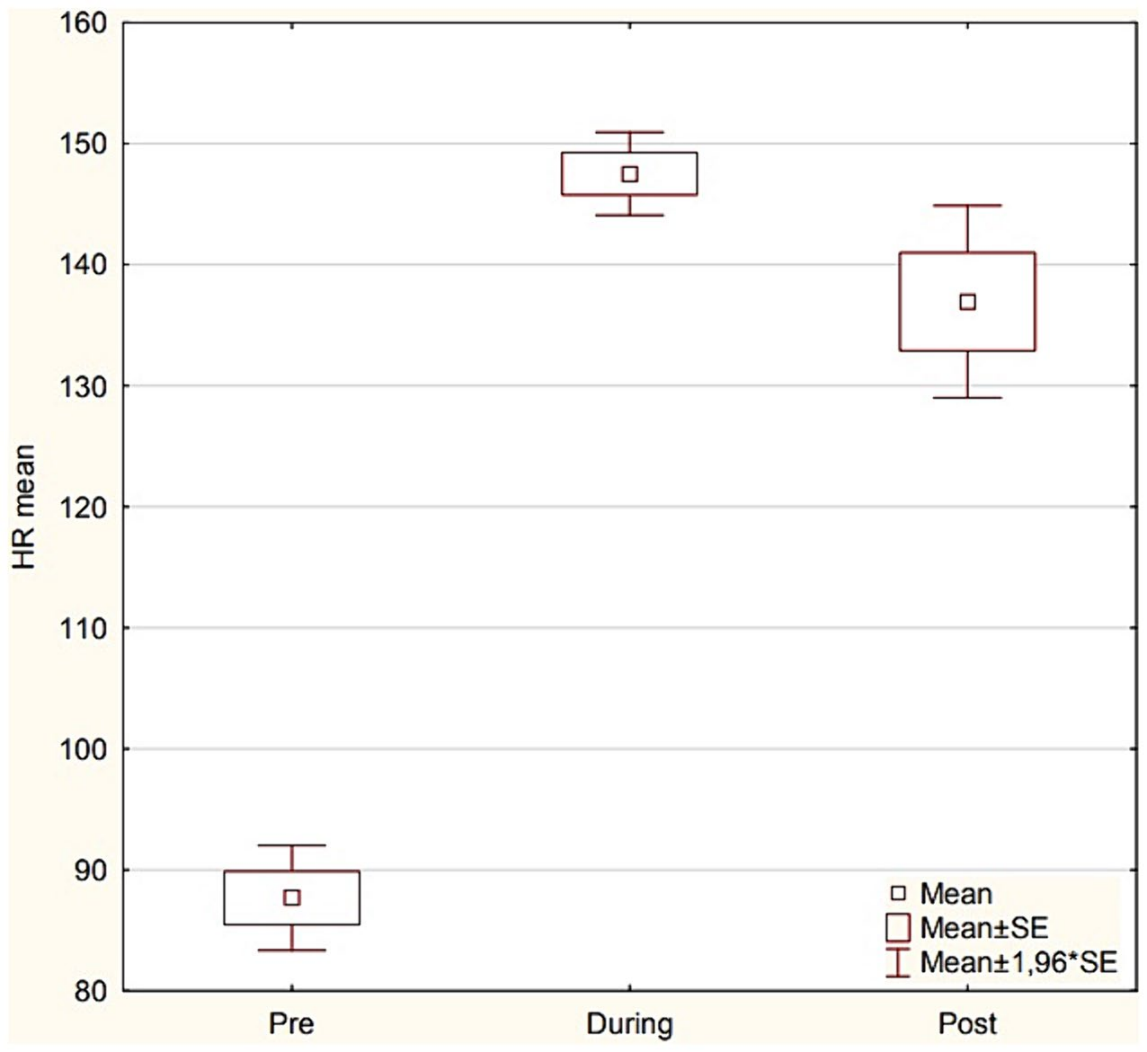
Results of HR mean according to study stage (Pre vs. During vs. Post).

From the group *n* = 75 the results of 17 firefighters were selected (Table 2; Figure 4). Analysis of selected parameters describing HRV changes was carried out, which are important from the authors’ experience: RMSSD, HF ms2, DFA α1 (Table 3).

**Table 2:** Heart rate analysis.

	Pre	During	Post	*p*
HR min	80.6 ± 12.9^#^	84.4 ± 13.0^#^	121.4 ± 22.9	< 0.001
Age < 28 yr	85.8 ± 9.0	82.4 ± 11.5	127.3 ± 24.7	
Age ≥ 28 yr	77.3 ± 13.8	86.1 ± 14.1	116.7 ± 20.9	
*p*	0.055	0.264	0.301	
BMI < 25	79.3 ± 12.1	84.3 ± 13.7	120.4 ± 20.1	
BMI ≥ 25	81.8 ± 13.11	84.6 ± 12.7	122.3 ± 25.5	
*p*	0.539	0.839	0.726	
Seniority <5 yr	86.3 ± 8.9	83.3 ± 11.2	128.3 ± 23.4	
Seniority ≥5 yr	76.4 ± 13.6	85.7 ± 14.9	114.5 ± 20.9	
*p*	0.021	0.566	0.099	
Activity (min) <38	79.7 ± 12.2	84.9 ± 12.5	122.3 ± 25.3	
Activity (min) ≥ 38	83.6 ± 13.7	84.0 ± 13.6	119.1 ± 16.2	
*p*	0.446	0.722	0.874	
HR max	93.9 ± 12.1*^#^	189.0 ± 13.1^#^	164.0 ± 23.4	< 0.001
Age < 28 yr	95.5 ± 10.1	189.4 ± 13.1	171.1 ± 22.9	
Age ≥ 28 yr	92.5 ± 13.2	188.7 ± 13.2	158.4 ± 22.8	
*p*	0.575	0.903	0.095	
BMI < 25	94.3 ± 12.1	189.2 ± 14.1	164.3 ± 23.3	
BMI ≥ 25	93.4 ± 12.2	188.9 ± 12.4	163.8 ± 24.1	
*p*	0.742	0.744	0.975	
Seniority <5 yr.	96.2 ± 10.1	189.1 ± 13.9	171.5 ± 22.7	
Seniority ≥5 yr	91.7 ± 13.2	188.9 ± 12.3	156.6 ± 22.3	
*p*	0.331	0.787	0.049	
Activity (min) <38	93.9 ± 9.6	182.3 ± 13.3	160.9 ± 23.1	
Activity (min) ≥38	93.3 ± 17.0	194.9 ± 9.7	172.2 ± 23.3	
*p*	0.845	<0.001	0.168	
HR mean	87.7 ± 12.6 *^#^	147.5 ± 15.2	136.9 ± 24.3	< 0.001
Age < 28 yr	89.2 ± 8.3	149.2 ± 16.3	143.7 ± 24.7	
Age ≥ 28 yr	86.1 ± 14.6	146.1 ± 14.2	131.6 ± 23.2	
*p*	0.368	0.606	0.108	
BMI < 25	85.1 ± 10.3	147.1 ± 15.3	136.5 ± 23.2	
BMI ≥ 25	89.3 ± 13.4	147.8 ± 15.2	137.3 ± 25.8	
*p*	0.203	0.814	0.714	
Seniority <5 yr	90.4 ± 8.1	148.5 ± 16.1	143.3 ± 23.6	
Seniority ≥5 yr	85.5 ± 14.8	146.4 ± 14.2	130.6 ± 23.9	
*p*	0.226	0.791	0.091	
Activity (min) <38	87.0 ± 10.7	147.9 ± 17.6	137.8 ± 25.6	
Activity (min) ≥38	89.2 ± 16.3	147.2 ± 12.9	134.7 ± 21.8	
*p*	0.728	0.610	0.805	

HR, heart rate; BMI, body mass index. *p < 0.05 at post-hoc analysis with during activity. ^#^*p* < 0.05 at post-hoc analysis with post activity.

**Table 3:** HRV analysis.

	Pre	During	Post	*p*
RMSSD	10.2 (8.0–11.5)*^#^	1.2 (1.0–2.6)	5.4 (1.0–7.1)	< 0.001
Age < 35 yr	10.5 (1.4–11.4)	1.2 (1.0–2.6)	1.0 (0.9–5.4)	
Age ≥ 35 yr	9.3 (8.2–12.4)	1.4 (0.95–3.0)	7.0 (3.7–8.3)	
*p*	0.630	0.847	0.092	
BMI < 25	10.9 (10.2–11.5)	1.3 (0.9–2.6)	3.9 (0.8–9.7)	
BMI ≥ 25	8.4 (1.3–11.9)	1.2 (1.0–3.5)	5.4 (1.1–7.1)	
*p*	0.291	0.615	0.763	
Seniority <7 yr	10.4 (1.3–11.6)	2.2 (1.1–3.6)	3.2 (0.9–6.7)	
Seniority ≥7 yr	10.2 (8.4–11.5)	1.1 (0.9–1.6)	7.0 (1.1–7.4)	
P	0.564	0.112	0.441	
Activity (min) <27	8.0 (1.3–11.2)	1.6 (1.1–2.6)	1.2 (1.0–7.4)	
Activity (min) ≥27	11.0 (9.3–12.0)	1.1 (0.9–3.0)	6.6 (1.0–7.1)	
*p*	0.083	0.248	1.0	
HF ms^2^	26.0 (12.0–29.0) *^#^ 0.00 (0.0–1.0)	4.0 (0.0–12.0)	< 0.001	
Age < 35 years	27.0 (0.0–29.0)	0.0 (0.0–1.0)	0.0 (0.0–4.0)	
Age ≥ 35 years	19.5 (12.5–34.0)	0.0 (0.0–2.0)	9.5 (4.0–15.0)	
*p*	0.961	0.847	0.229	
BMI < 25	32.5 (27.0–40.0)	0.0 (0.0–1.0)	4.0 (0.0–27.0)	
BMI ≥25	13.0 (0.0–28.0)	0.0 (0.0–3.0)	4.0 (0.0–12.0)	
*p*	0.044	0.725	0.802	
Seniority <7 yr	24.5 (0.0–31.5)	0.5 (0.0–3.5)	2.0 (0.0–15.5)	
Seniority ≥7 yr	28.0 (13.0–29.0)	0.0 (0.0–1.0)	8.0 (0.0–12.0)	
*p*	0.500	0.470	0.961	
Activity (min) <27	12.0 (0.0–28.0)	0.0 (0.0–1.0)	0.0 (0.0–12.0)	
Activity (min) ≥27	27.0 (18.0–32.5)	0.0 (0.0–2.0)	8.0 (0.0–14.5)	
*p*	0.210	0.847	0.773	
DFA α1	0.98 (0.93–1.11)	0.96 (0.88–1.09)	0.94 (0.85–1.04)	0.662
Age < 35 yr	1.06 (0.93–1.11)	0.97 (0.90–1.09)	0.92 (0.85–1.01)	
Age ≥ 35 yr	0.96 (0.92–1.05)	0.88 (0.79–1.07)	1.02 (0.89–1.07)	
*p*	0.736	0.229	0.361	
BMI < 25	0.93 (0.91–0.98)	0.99 (0.87–1.19)	0.84 (0.70–0.89)	
BMI ≥25	1.08 (0.94–1.14)	0.96 (0.88–1.06)	1.01 (0.93–1.10)	
*p*	0.078	0.651	0.048	
Seniority <7 yr	1.07 (0.87–1.11)	1.02 (0.93–1.13)	0.90 (0.82–0.97)	
Seniority ≥7 yr	0.98(0.93–0.99)	0.88 (0.81–0.98)	1.03 (0.94–1.10)	
*p*	0.961	0.075	0.194	
Activity (min) <27	0.93 (0.83–1.08)	0.98 (0.90–1.13)	0.92 (0.84–1.01)	
Activity (min) ≥27	1.02 (0.96–1.13)	0.88 (0.79–1.03)	0.98 (0.89–1.09)	
*p*	0.194	0.075	0.413	

^*^p < 0.05 at post-hoc analysis with during activity. ^#^*p* < 0.05 at post-hoc analysis with post activity.

Results with artifacts that make it impossible to generate descriptive parameters HRV from raw RR records were not taken into account for statistical calculations. The authors have raw records of RR min, RR max, RR mean, time of the study, but these results were not included because the separation was not consistent with the purpose of the study (HRV changes).

Interaction plots with results from Table 2 are shown in Supplementary Figure S1.

There is a significant difference between Pre HR min and Post HR min, and between During HR min and Post HR min (adjusted *p*-values <0.05). There is a significant difference between Pre HR max and During HR max, and between Pre HR max and Post HR max (adjusted *p*-values <0.05). There is also a significant difference between During HR max and Post HR max (adjusted *p*-value <0.05). There is a significant difference between Pre HR mean and During HR mean, and between Pre HR mean and Post HR mean (adjusted *p*-values <0.05).

There is a significant difference between Pre RMSSD and During RMSSD, and between Pre RMSSD and Post RMSSD (adjusted *p*-values <0.05). There is a significant difference between Pre HF ms2 and During HF ms2, and between Pre HF ms2 and Post HF ms2 (adjusted *p*-values <0.05) (Table 3). In the comparative statistical analysis of HR variability, statistically significant differences were shown in terms of employment experience in Pre stage group (*p* = 0.021) and Post stage group (*p* = 0.049) and activity in During stage group (*p* < 0.001) (Table 2).

In the comparative statistical analysis of HRV variability, statistically significant differences were shown in terms of BMI in Pre stage group (*p* = 0.044) and Post stage group (*p* = 0.044) (Table 3).

The effort with the band was well tolerated by the firefighters. Firefighters who answered “YES” were asked to justify their answers:

1. Too small band circumference – pressure was felt and it absorbed attention.
2. Pinching sensation at the height of the sternum.

## 4 Discussion

One of the parameters of HRV analysis is DFAα1, which correlates quite well with VO2max, which is the gold standard for assessing performance. The choice of the Polar H10 to collect the parameters of a firefighter in the smoke chamber is supported by current research, in which the Polar H10 is a sensor that is more accurate for measurement, at a relatively low cost, moreover, its size and mounting on the chest does not adversely affect the planned task. This type of solution is used in many sports (13).

In our own study, the sensor location was well tolerated by 98% of firefighters – XXL size was used for all of them, which, according to the manufacturer’s technical data.

The measurement of HRV parameters is used in many clinical conditions, in diagnosing the condition of patients after COVID-19, after cardiac ischemia, complications of sepsis, multi-organ failure. Asarcikli followed a group of post-COVID-19 patients, analyzing autonomic function using HRV indices, taking into account the root mean square of consecutive RMSSD. Increased parasympathetic activity has been demonstrated in patients with a history of COVID-19, which may be related to autonomic imbalance. The same parameter analyzed in our own study. RMSSD has a wide range of clinical use, in 2022 the authors studied changes in people after COVID-19, instead we used the reading in a population of healthy people subjected to significant occupational strain (physical and mental) (14, 15).

Shaffer et al. divide measurements into short-term (minutes) and long-term (≥24 h). The authors point out that long-term HRV records can predict health outcomes: heart attack, stroke, and all-cause mortality. In our own study, we made short-term measurements. Our population consists of healthy and fit people, and the assessment concerned adaptation to conditions of exercise, increased temperature and stress. Short-term HRV measurements are also described by other authors (16, 17).

An interesting observation of firefighters in conditions of increased temperature in protective clothing was made by Dwornik et al. This study did not include an HRV analysis, but the temperature burden of protective clothing (Nomex) is similar to the conditions created for firefighters in our own study. A specialized outfit protects against fire, but can potentially be dangerous for firefighters in action. It is associated with increased internal body temperature, it causes rapid breathing, increased heart rate, blood pressure, significant sweating and loss of essential electrolytes. The study was based on the analysis of several parameters: temperature, weight, heart rate, blood pressure, and saturation (18).

Schneider et al. pay attention to changes in HRV observed for psychological reasons. The authors observed changes in ANS function for a variety of psychiatric disorders, including post-traumatic stress disorder (PTSD)—a lower HRV compared to the control group. The observations concerned the following parameters: root mean square of successive differences (RMSSD), spectral components of low frequency (LF) and high frequency (HF). One of the objectives of own study was to observe differences in HRV under the influence of the stress factor in a closed, smoky smoke chamber. HRV analysis for behavioral changes was also studied by Zhu (19, 20).

Heart rate variability (HRV) analysis can be a useful tool for detecting underlying heart problems and even general health problems. Currently, such analysis is usually carried out under controlled or semi-controlled conditions. Because many common HRV measures are sensitive to data quality, manual artifact correction is common in the literature, both as an exclusive method and in addition to various filters. With the proliferation of personal monitoring devices that enable continuous HRV analysis, opportunities for HRV analysis in a new setting are opening up. In our own analysis, due to the complicated procedure and dynamic scenario of effort for each firefighter, there was also a large number of artifacts, the number of which in several cases led to the decision not to include the result in the analyzed group (21).

Pham et al. point out the technical aspect and difficulty in obtaining HRV, and the process of obtaining the parameter is complex and therefore presents a challenge for users who may not have adequate general knowledge to reliably obtain HRV indicators. This is confirmed by our own study, a time-consuming process was performed between the raw RR and the HRV parameter (22, 23).

In the literature on the subject in the population of firefighters, it is worth mentioning 2 more studies. Saari et al. (24) investigated the differences between resting and post-exercise state simulated on the pitch. Differences in several parameters were observed: average heart rate reserve (HRRes), heart rate variability from rest to post-exercise stage (LnRMSSDRest-Post), recovery rate (HRR60) was measured 60 s after exercise.

Zhu studied, that HRV analysis plays an important role in examining and detecting emotions. In this aspect, the study meets our expectations. We wanted to visualize changes in HRV due to physical load (fatigue), but also in emotional, negative changes (stress and heat stress) (25).

Marciniak used a far infrared sauna as a post-fire simulation to address carcinogen issues. Inability of the ANS to fully recover after calling for help, manifested by increased activity of the sympathetic nervous system and delayed reactivation of the parasympathetic nervous system (26). Both of these simulation studies on group of firefighters are partially consistent with our own methodology – reaction to physical exercise (pre-post), heat stress.

Numerous artifacts occurred in measurements according to our methodology. As Królak points out, HRV testing is sensitive to artifacts even in controlled conditions (in our study, a controlled smoke chamber). As the authors point out, it is possible to manually correct changes or use filters, which is consistent with our research. We used special software to interpret the results, which was then manually corrected during a long process (27).

### 4.1 Strengths and limitations of the study

Our study had some limitations. A small group enrolled in the study. The authors had no influence on the selection of the group in terms of positions held in the State Fire Service (rescuer, driver, commander). A breakdown by position could provide additional inferences about the result correlated with the daily duties of firefighters. In practice, firefighters who are in the group of rescuers most often search rooms affected by fire, evacuate injured people, and this group is most often exposed to the burden provided by the smoke chamber. The results of some of the firefighters who participated in the study were not included in the analysis (*n* = 21) due to a large number of artifacts in the heart rate recording, or connectivity problems and incomplete recording of the entire exercise as intended by the study (stage 2). The smoke chamber is entirely made of metal elements: walls, grates, floor, ceiling, which in many cases disrupted the connection between the sensor and the mobile device with software.

The study on HRV among professional firefighters during smoke chamber testing embodies several notable strengths that bolster its scientific value and practical relevance. Firstly, the innovative study design is a significant asset, assessing the ANS’s reactivity in a setting that closely mimics the high-stress environments firefighters routinely encounter. This approach not only enhances the applicability of the findings to firefighter training and health monitoring but also contributes to the existing body of knowledge by providing a detailed analysis of physiological responses under simulated yet realistic conditions. Additionally, the comprehensive data collection across various physical activities and rest periods, along with the inclusion of a diverse and substantial sample size of 96 firefighters, ensures an analysis that enhances the generalizability of the results to a broader firefighter population.

Despite these strengths, the study faces several limitations that could impact the interpretation and application of its findings. Technical challenges were evident, particularly with the connectivity and data accuracy from the HRV monitoring devices in the metal-constructed smoke chamber, potentially leading to data loss or inaccuracies. Moreover, the study primarily focuses on physiological indicators without an extensive evaluation of the psychological impacts of stress, which are vital for a holistic understanding of firefighter well-being. Additionally, the apparent exclusion of female firefighters from the study sample might limit the findings’ applicability across the entire firefighter workforce, as gender can influence physiological responses to stress and training.

## 5 Conclusion

The study on HRV in firefighters undergoing smoke chamber tests provides vital insights for enhancing firefighter health and safety protocols. HRV measurements emerge as crucial indicators of a firefighter’s capacity to handle the intense stress and physical demands of their role. Implementing HRV monitoring in routine health assessments could identify those at greater risk of stress-related health issues, facilitating tailored training and recovery plans that may reduce cardiovascular risks and improve overall health outcomes.

Additionally, the research stresses the need for a comprehensive training approach that incorporates both physical endurance and stress management. Developing training programs that boost the autonomic nervous system’s regulation could significantly enhance stress resilience among firefighters.

Overall, this study establishes HRV as a valuable tool for predicting and improving firefighters’ health and operational readiness. Future research should focus on refining data collection techniques and broadening the participant base to strengthen the applicability of HRV assessments in fire services, ensuring better safety and effectiveness for all firefighters. The difficulty in the implementation of the methodology is related to the construction of the stationary smoke chamber (metal structural elements, sensors, cameras, installations), which hinders Bluetooth connectivity.

## Data availability statement

The datasets presented in this article are not readily available because data of uniformed service officers. Requests to access the datasets should be directed to lukasz_dudzinski@o2.pl.

## Ethics statement

The studies involving humans were approved by John Paul II University in Biała Podlaska, Poland. The studies were conducted in accordance with the local legislation and institutional requirements. The participants provided their written informed consent to participate in this study.

## Author contributions

ŁD: Conceptualization, Data curation, Funding acquisition, Investigation, Methodology, Project administration, Resources, Software, Supervision, Validation, Visualization, Writing – original draft, Writing – review & editing. ŁC: Formal analysis, Supervision, Writing – review & editing. MP: Formal analysis, Supervision, Validation, Writing – review & editing.
