# Supplementary figures and images for: Assessment of parameters reflecting the reactivity of the autonomic nervous system of Polish firefighters on the basis of a test in a smoke chamber
Source: Front Public Health. 2024 Jul 19;12:1426174. doi: 10.3389/fpubh.2024.1426174 (PMC11297351; doi:10.3389/fpubh.2024.1426174)

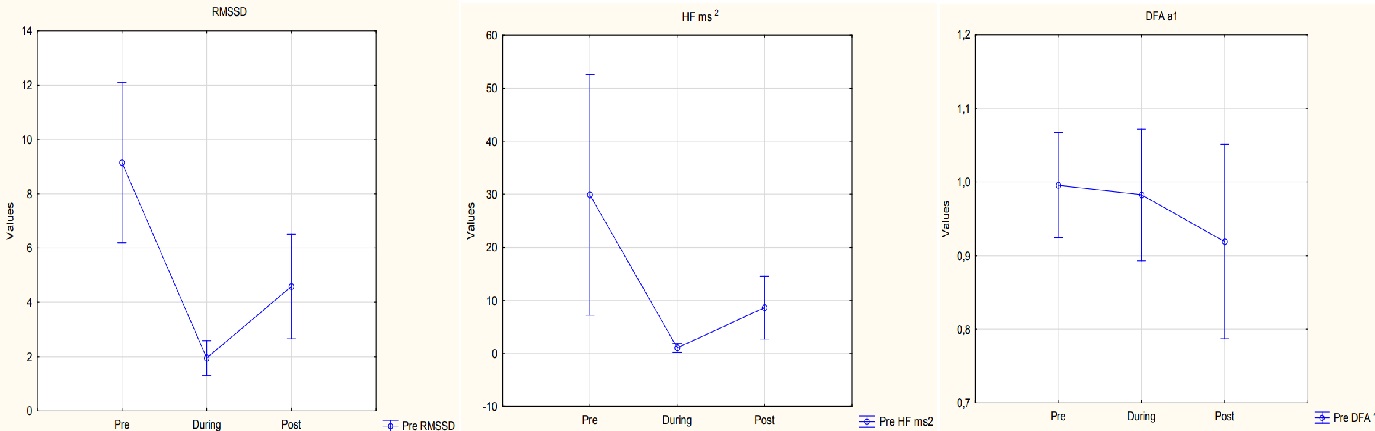

Supplement: Supplementary file 1 [file Image_1.jpeg]
